# Anti-Inflamm-Ageing and/or Anti-Age-Related Disease Emerging Treatments: A Historical Alchemy or Revolutionary Effective Procedures?

**DOI:** 10.1155/2018/3705389

**Published:** 2018-02-08

**Authors:** Carmela Rita Balistreri

**Affiliations:** Department of Pathobiology and Medical and Forensic Biotechnologies, University of Palermo, Palermo, Italy

## Abstract

The “*long-life elixir*” has long represented for humans a dream, a vanity's sin for remaining young and to long survive. Today, because of *ageing population phenomenon*, the research of antiageing interventions appears to be more important than ever, for preserving health in old age and retarding/or delaying the onset of age-related diseases. A hope is given by experimental data, which evidence the possibility of retarding ageing in animal models. In addition, it has been also demonstrated in animal life-extending studies not only the possibility of increasing longevity but also the ability to retard the onset of age-related diseases. Interestingly, this recent evidence is leading to promise of obtaining the same effects in humans and resulting in benefits for their health in old ages. In order to achieve this goal, different approaches have been used ranging from pharmacological targeting of ageing, basic biological assays, and big data analysis to the recent use of young blood, stem cells, cellular, genetic, and epigenetic reprogramming, or other techniques of regenerative medicine. However, only a little fraction of these approaches has the features for being tested in clinical applications. Here, new emerging molecules, drugs, and procedures will be described, by evidencing potential benefits and limitations.

## 1. Introduction

The current goal of ageing's researchers is both to understand the biology of ageing and the *related inflamm-ageing* and to translate the potential scientific insights obtained into effective interventions, in order to improve the health in old ages and to retard and/or to delay the onset of age-related diseases (ARDs) [1]. Thus, antiageing and anti-inflamm-ageing interventions and the parallel health promotion for old people represent the very recent interests not only of the entire scientific community but also of all institutions/organizations from the Western countries and particularly from EU countries [2]. These last are recommending *health promotion programmes,* such as *EU Project Pro-Health* 65+, which engages ten EU member states [2]. Cotemporally, this growing research is becoming a very business for several biotechnological companies, which are promoting a growing number of therapeutic approaches, ranging from oxidant drugs, hormones, vitamins, and diet supplements to various aesthetic drugs and techniques [3]. In fact, these approaches are particularly costly for gullible patients in searching of well being and abused by a carefully organized marketing involving tacit complicity of doctors, laboratories, and companies [3]. However, the major number of these treatments until now constitute more a *palliative care* than a very “*long-life elixir*,” which has long represented for humans a dream, a vanity's sin for remaining young and to long survive [3]. This leads to the extemporaneous consideration that the way, for developing effective antiageing and anti-inflamm-ageing treatments in humans, is distant. Despite this, there is among the ageing's researchers the optimistic hope in identifying successful treatments and approaches for humans too. It derives from the increase of lifespan observed in short-lived model organisms (i.e., yeast, worm, flies, mice, and rats) which undergone to various genetic, dietary, and pharmacological interventions [4–7]. In addition, some of these treatments have also demonstrated to retard the onset of ARDs and, consequently, to extend the health-span (i.e., the length of time one lives in good health), as evidenced in 2014 by Kennedy and coworkers [8]. Thus, it is possible to affirm that this evidence in animal models leads to the promise to develop effective life-extending interventions in humans too. Accordingly, new treatments are emerging. Here, some of these interventions will be described and discussed, underlying the benefits and limitations. Before their dissertation, a brief clarification of some concepts will be pointed in the initial paragraphs. This will facilitate the understanding of arguments reported not only for the ageing's researchers but also for clinicians, since the author's message is to offer a concrete “*food for thought*” about ageing and new effective therapeutic strategies to counteract, to retard, or to delay ARDs, such as the cardiovascular diseases (CVDs), the first very problem of our populations. In fact, the concepts stressed, here, are fruit of considerations derived by expert opinion on the findings from author's studies on ageing, ARDs (particularly CVDs) and inflammation.

### 1.1. Aspects to Point about Antiageing Treatments: From Definition to Eventual Use and New Strategies for Their Identification

Today, the lack of a clear consensus on what exactly constitutes an *antiageing treatment* still exists. Initially, it imagined to define as antiageing treatment the approach or drug able to counteract the primary cause of ageing, which still remains to identify and may be consequently considered only a very abstraction and not a reality. Recently, Gems has stressed this concept and has suggested the necessity to propose a more pragmatic and realistic definition of *antiageing treatment* [9]. Precisely, he has proposed to define with the term *antiageing treatment* as any preventative approach able to reduce late-life disease, since new evidence considers the senescence (i.e., ageing) a disease's syndrome [10]. In fact, Gems's definition is essentially based on the emerging concept of ageing to consider it as set of pathologies, some of which provoke mortality [9]. Thus, Gems has suggested that the key to understanding ageing is both to better characterize the ARDs (including diabetes, CVDs, neurodegenerative disorders, and cancer) and discover their original causes. As a result, this definition would include all preventative approaches aimed for ARDs [9]. In addition, its use would help its translation, since it would change the weight of medical practice, and in the specific case, the opening to preventative approaches [9]. For examples, the cardiovascular polyp ill treatment, proposed as antiageing treatment for CVDs, could establish a medical practice that extends it to antiageing preventive interventions, such as dietary restriction mimetics [11].

The suggestion from Gems is the result of the symposium “*Interventions to Slow Aging in Humans: Are We Ready*?” held on October 8–13, 2013, in Erice (Sicily) and organized by Valter Longo, Luigi Fontana, and Donald Ingram [9]. In addition, it does not pretend to refute the initial definition of antiageing treatment, but it emphasizes the necessity of clearing the concept and stabilising a common consensus on a standardized definition of antiageing treatment and likely proposing novel strategies for achieving it. Accordingly, Saraswat and Rizvi [12] have recently proposed that one of the most promising approaches for a successful antiageing strategy includes the activation of *adenosine monophosphate-dependent protein kinase (AMPK)*. They have also suggested that another strategy may involve the activation of plasma membrane redox system (PMRS), and the consequent development of molecules able to activate or inhibit nutrient and energy-sensing molecular pathways, such as *mTOR*, *IGF-1*, *AMPK*, and *sirtuins* [12]. Other new antiageing (or precisely anti-ARD, that is, CVDs) strategies have been also proposed by our group. In particular, *very innovative frameworks* for developing therapeutic interventions for some ARDs, such as CVDs and neurodegenerative diseases, have been suggested by our group [13–19]. They are based on the relevant concept that each individual is the result of the sophisticated interplay between environmental factors and its genome, trascriptome, proteome, metabolome, microbiome, epigenome, and exposome. Thus, it is necessary to perform a more complex combination of investigations based on genetic, transcriptomic, proteomic, metabolomic, microbiomic, and epigenetic evaluations, for obtaining interesting data in the study of these diseases. In addition, computational investigations are also recommended, as well as collecting environmental and biometric data and medical/scientific/health care records [13–19]. Thus, we have suggested that the integration of all data, obtained in this large panel of investigations, and their elaboration might lead to the development of appropriate agonists, antagonists, and inhibitors of specific signaling disease pathways, which might be used in a near future as *personalized treatments* for these diseases, facilitating their management and outcome [13–19].

### 1.2. An Emerging Class of Antiageing Treatments, “the Pharmacological Targets of Ageing”: A Brief Description

**Figure 1:**
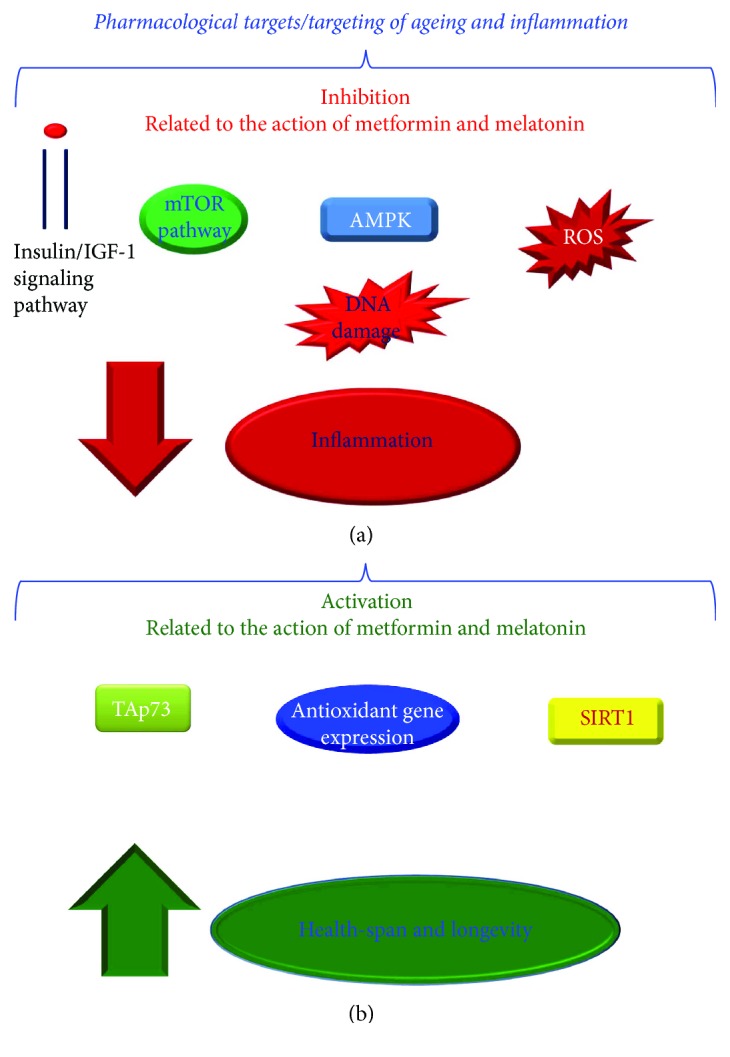
(a and b) Pharmacological targets/targeting of ageing and inflammation. In (a and b), examples of ageing and inflammatory target pathways. In particular, in (a), it describes the inhibitor actions of metformin and melatonin on ageing/inflammatory pathways. In (b), the activation of some ageing/inflammatory pathways for counteracting the ageing and inflammatory effects and for contributing to health-span and longevity.

**Figure 2:**
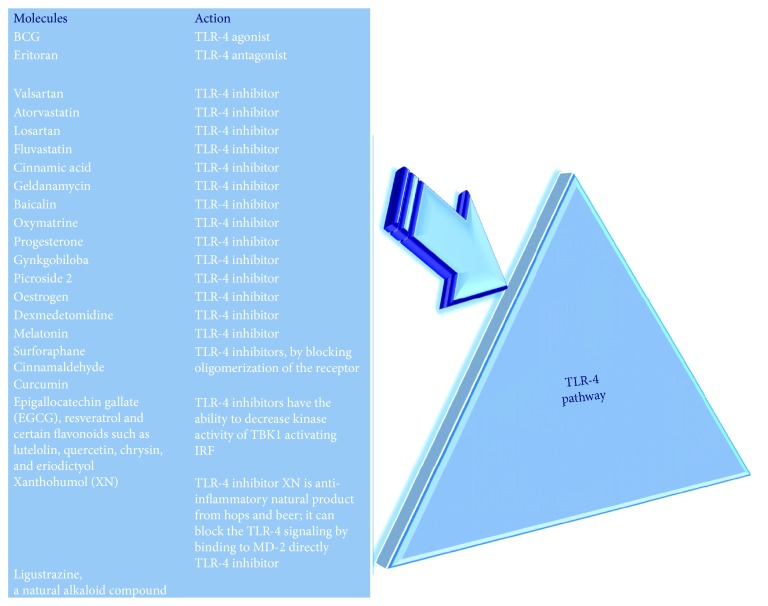
The TLR-4 pathway and a panel of agonist, antagonist, and inhibitor molecules.

A plethora of genes, molecules, and pathways able to modulate the ageing or better the ARDs is emerging from several studies, in both animal models and humans, as recently stressed by our and other groups [4–6, 13–19]. They have been suggested as potential targets for antiageing (anti-ARD) treatments and are likely leading to development of that class of antiageing treatments defined as *pharmacological targets of ageing*, while the approach used has been defined as *pharmacological targeting of ageing* (of ARDs) (see Figures 1(a) and 1(b)) [4–6, 13–19]. Among the pathways detected as potential antiageing targets, it is possible to identify in first position *the immune inflammatory pathways*. Their relevant weight derives from several data, which have strongly suggested them as the promoters of ageing (senescence) and ARDs, as also evidenced in our studies [13–19]. This is in agreement to strong concept emerged by a very large number of ageing and ARD studies that *sterile inflammation* and the consequent systemic chronic inflammation at low rate (i.e., *inflamm-ageing*) [20–25], related to the release of the so-called *senescence-associated secretory phenotype* (SASP) [19, 26, 27], are the primary mechanisms of ageing and the pathophysiology of all ARDs [20–27]. Here, it reports a typical example of these pathways, and precisely, the *Toll-like receptor-4 (TLR-4) signaling pathway* (see Figure 2), [13, 28], which the emerging evidence places in the center (constituting it a network with other evolutionally conserved pathways) of molecular mechanisms involved in senescence (ageing) of all cells, tissues, organs, and systems and the related ARDs [13–19, 25, 28–32], as well as in those associated with the host defence against Gram-negative bacteria, viruses, fungi, and mycoplasma [33]. Thus, TLR-4 pathway shows a double role in human tissues, which is modulated and influenced by the context of each tissue, organ, and system and particularly by the conditions of their microenvironment, as we have recently suggested in a model proposed for aorta homeostasis and disease [13]. The growing interest on TLR-4 pathway is leading some biotechnology companies to develop various drugs (i.e., agonists, antagonists, and inhibitors), ranging from proteins to metal ions, as pharmacological treatments to modulate ageing and ARDs, such as CVDs (see Figure 2). Their action has shown that both the TLR-4 activation and inhibition may have beneficial effects in various conditions, reducing tissue immune inflammatory responses (as amply quoted in [13, 19, 28]). However, the biological effects mediated by TLR-4 modulators seem to depend on several factors. They include the timing of administration during the various stages of ageing or disease onset and progression and the age and genetic background of animal models used during experimentation (as amply quoted in [13, 19, 28]). Regarding this consideration, we have stressed in a recent review [28] that there are age-related defects in TLR-4 function and expression, as reported in human studies. However, these studies did not lead to conclusive data, being limited by the heterogeneity of epidemiological and laboratory methods [28]. This might also be valid for the animals used as study's model. Furthermore, current evidence demonstrates that the TLR-4 function and expression are modulated not only by genetic variants and haplotypes [28] but also by environmental factors. Accordingly, diet (quoted in [13]), mite allergens (quoted in [13]), air pollution (quoted in [13]), and their cross-interaction with microbiota (quoted in [13]), which may remain in a healthy state or show alterations (i.e., dysbiosis and consequent endotoxemia associated with age or obesity) (quoted in [13]), and consequent epigenetic changes (quoted in [13]), have been significantly associated with TLR-4 functional alterations and modified tissue levels. Consequently, many limitations and concerns emerge. In addition, another relevant limitation derives from the type of investigation. In this specific case, TLR-4 agonists and antagonists have been mainly tested on cell cultures and animal models (quoted in [13]). Thus, at moment, only preclinical data exist, but not human trials. This leads to affirm that the encouraging data obtained until now in animal models may not be likely confirmed in humans. In fact, there are many concerns in this field. The first concern comes from the real action mediated by TLR-4 agonist and antagonist molecules, which is amply questioned. A large number of researchers claim that only a little subset of them is able to activate or inhibit directly the TLR-4 pathway. The limitation stays in their structural diversity that likely implies a promiscuity in their TLR-4 binding capacity, without specificity of action, and rather a pleiotropic effect on different receptors (quoted in [13]). Similar considerations may also be valid for small natural molecules, such as curcumin, cinnamaldehyde, and sulforaphane, which have been reported to mediate the anti-TLR-4 dimerization effects, as well as for resveratrol and some flavonoids (such as EGCG, lutelolin, quercetin, chrysin, and eriodictyol), reported to inhibit TBK1 kinase activity (a downstream TLR-4 signaling kinase), resulting in a decreased IRF3 activation (a TLR-4 signaling adaptator) and target gene expression (quoted in [13]). Thus, these observations support the idea that further studies are imperative for clinical implementations in the near future of these molecules. On the other hand, it has been suggested in the previous paragraph that the solution can be in putting together several types of investigations with the evaluations of TLR-4 modulators to facilitate the development of *personalized treatments* for ageing and ARDs, such as CVDs (as below reported).

## 2. Metformin: The “Miraculous” Drug for Counteracting Ageing and ARDs

Recently, *the metformin*, one drug normally used in diabetes therapy, is emerging as antiageing factor (quoted in [33, 34]). Studies on model organisms have, indeed, demonstrated that metformin has the ability to extend the lifespan (quoted in [33, 34]). The potential mechanisms used have been largely discussed and include the following: (a) the reduced insulin and IGF-1 signaling; (b) the inhibition of mTOR; (c) reducing the levels of reactive oxygen species (ROS); (d) lowering inflammation, (e) reducing DNA damage, and (f) the activation of AMPK (quoted in [33, 34]). Its effect on AMPK has attracted particular attention (see Figures 1(a) and 1(b)). It also appears to modulate the intracellular mechanisms of caloric restriction, long recognized as the first intervention capable of extending the lifespans in animal models, but not feasible for widespread implementation in humans (quoted in [33, 34]). This, among other factors, has led to use metformin, being described as a caloric restriction mimetic (CRM) (quoted in [33, 34]). These encouraging results have attracted the attention of other groups to perform further investigations in a large panel of ARDs. The data obtained have demonstrated that metformin also shows antitumor, cardiovascular protective, and neuroprotective effects (quoted in [33–36]). Interestingly, this evidence has attracted the attention of the Campbell group in 2017 [35] that performed a meta-analysis for evaluating the effect of metformin on all-cause mortality or diseases of ageing relative to nondiabetic populations or diabetics receiving other therapies with adjustment for disease control achieved [35]. They obtained very promising data. Precisely, they observed that metformin is not only significantly able to reduce all-cause mortality in diabetic cases taking it than nondiabetics but also in did diabetics taking metformin compared to diabetics receiving nonmetformin therapies, such as insulin or sulphonylurea. In addition, they detected that metformin users also show a reduced risk for cancer and CVDs compared to nondiabetics or diabetics receiving nonmetformin therapies or insulin [35]. Thus, they concluded that metformin could represent an effective extending life and health-span drug, by acting as an antiageing factor [35]. This potentiality of metformin has encouraged the Wang group to propose potential models for explicating the mechanisms and the pathways involved [34].

However, many questions still remain unresolved. For example, it is not detected whether these potential indications of metformin can be also observed in nondiabetics and whether genetic factors can influence the effects of metformin in each individual. Thus, it is necessary to clarify these aspects, by performing not only further research basic investigations but also clinical trials.

## 3. Melatonin, the Molecule Regulating Human Physiological Rhythm as Antiageing Treatment

Another emerging antiageing molecule is the *melatonin (N-acetyl-5-methoxytryptamine)*, an indoleamine molecule highly and generally identified in the major number of plant and animal organisms, including human [37]. It is synthesized from the essential amino acid l-tryptophan thanks the action of four enzymes [37]. In vertebrates, including human, melatonin is recognized as a secretory product of the pineal gland. However, in these organisms, it and four enzymes also are the physiological cell components of other tissues, including the retina, skin, immune system, gastrointestinal tract, and reproductive tract (amply quoted in [38]). Here, melatonin is expressed at different levels, and it shows a higher density of expression within the membranes and the mitochondria, where it mediates several functions: interacts with lipids, stabilizes all cellular membranes, reduces lipid peroxidation, and increases ATP production as evidenced by García and coworkers in 2014 [39]. In addition, it evocates several beneficial effects: antioxidant and free radical scavenging capacity, that consent to melatonin to protect proteins and mtDNA from oxidative stress; capability of penetrating all morphophysiological barriers and entering all subcellular compartments due to its amphiphilic nature, that permit to melatonin to modulate a diverse number of physiological processes via different mechanisms; capacity to active a broad spectrum of molecular pathways, including particularly the sirtuins, such as SIRT1, through both receptor-dependent and receptor-independent signaling pathways; a powerful direct free radical scavenger ability against ROS and reactive nitrogen species (RNS); capacity to increase the activity of endogenous antioxidant enzymes; and anti-inflammatory properties (see Figures 1(a) and 1(b)) (amply quoted in [38–40]). This last action consents it to attenuate tissue damage under a variety of ARD conditions (amply quoted in [38–40]). Some of the anti-inflammatory properties are principally related to SIRT1 activation. Accordingly, melatonin has shown, in apolipoprotein E-deficient mice, the capacity to decrease the impairment of endothelial damage, the loss of SIRT1 and endothelial nitric oxide synthase, and the p53 and endothelin-1 expression [41]. In addition, melatonin has been also observed to confer a cardioprotective effect against myocardial ischemia-reperfusion injury, by reducing oxidative stress damage via activation of SIRT1 signaling in a receptor-dependent manner [42]. Melatonin treatment also seems to reduce cerebral ischemia-reperfusion injury in mice, by reducing ischemia-reperfusion-induced mitochondrial dysfunction through the activation of SIRT1 signaling and the attenuation of mitochondrial oxidative damage [43].

Recently, it has been also demonstrated that the production of melatonin within 24 hours is significantly higher in young animals than in old organisms, including humans. This has suggested a potential association between the loss of melatonin and the signs of ageing (amply quoted in [38]). In addition, in animal models, it has been observed that the rhythm of melatonin can be substantially preserved during ageing by restricting food intake (amply quoted in [38]). This has suggested that food restriction could mediate some of its beneficial physiological effects through its ability to sustain pineal activity in old age (amply quoted in [38]). Food restriction presumably conserves the melatonin rhythm in part, because it prevents the reduction in pineal-adrenergic receptors normally reported in old rats (amply quoted in [38]). The loss of melatonin in the elderly may lead to disorders of circadian rhythms that reflect in an increase of systemic and tissue inflammation and in the evocation of both senescence and ARDs (amply quoted in [38]).

The actions described above may elucidate, at least in part, its protective ability to delay the deleterious effects of ageing and a variety of ARDs, such as Parkinson's disease, Alzheimer's disease, ischemia-reperfusion, and sepsis (amply quoted in [36, 38, 40]). They are leading several companies to develop melatonin hybrids, as potent antiageing drugs. Furthermore, the use of foods rich of melatonin is recently recommended. Of course, both experimental basic investigations and clinical trials are necessary for its future clinical applications (amply quoted in [36, 38, 40]).

## 4. Stem Cells as Therapeutic Agents for Ageing and ARDs?

Recently, the clinical application of stem cells, as therapeutic agents in ageing and ARDs, is acquiring a very consideration from the entire scientific community [44, 45]. Stem cell therapy might, therefore, constitute a very solution for both delaying/retarding ageing processes and the onset/progression of ARDs and permitting to expand the survival of individuals. The hope also is of applying it in a near future as preventive treatment in susceptible ARD individuals [44, 45].

Stem cells are defined as undifferentiated cells with the potential to renew themselves and to differentiate into any other specialized cell of the human body and, therefore (potentially and theoretically), to create any tissues or organs ([17, 46]). Under specific conditions, stem cells can, indeed, differentiate into diverse populations of mature and functionally specialized cellular types. To date, the following classes of stem cells are recognized: (a) *totipotent cells*, having the capacity to differentiate into embryonic and extra embryonic cell types, thereby generating the entire organisms, even if this capacity is limited to cells produced by the first few divisions after fertilization; (b) *pluripotent stem cell types*; and (c) *adult multipotent/unipotent stem cells*, which can only differentiate into several closely related cell types ([17, 46]). A variety of cellular types has been and is currently used in cell-based therapy, including *embryonic*, *multipotent stromal*, *induced pluripotent*, and *adult stem cells* ([17, 46]). By analysing the role of all stem cells as therapeutic antiageing (and particularly anti-ARD) agents, the enthusiasm of researchers has been reduced by diverse concerns related to the detection of various serious limitations (quoted in [17, 36, 46]). In fact, diverse undesirable factors have been evidenced, including firstly the teratogenic and tumorigenic potential of these cells on the recipient organisms and the immune reactivity, which consequently affect the safety of the treatments, but also their reduced success related to the inadequate dose administration, the imprecise phenotypic profile of cells used for the treatments, their biological age and senescent status, and the inappropriate administration ways and methods used (quoted in [17, 36, 46]). Thus, ulterior studies are certainly needed to convert the research data in clinical applications, from research basic investigations to preclinical and clinical studies.

## 5. Cellular Reprogramming and Young Blood as Potential Antiageing Therapies?

An optimistic help in the development of antiageing treatments might derive from *Regenerative Medicine* (RegMed), a branch of *translational medicine* (amply quoted in [36, 46, 47]). It arises from a necessity of eliminating the gaps existing between the increased risk of disease and the decreased capacity of the major number of human tissues and organs spontaneously to regenerate and to respond to insults and damages in the setting of ageing (amply quoted in [36, 46, 47]). RegMed is, indeed, as interdisciplinary field of research and clinical applications focused on the repair, replacement, or regeneration of cells, tissues, or organs to restore impaired function resulting from any causes, including congenital defects, diseases, trauma, and ageing (amply quoted in [36, 46, 47]). For this reasons, RegMed is suggested as the way “to improve the health and quality of life by restoring, maintaining, or enhancing functions of tissue and organs” (amply quoted in [36, 46, 47]). For achieving these objectives, RegMed uses a combination of several approaches, including (but not limited to) the use of soluble molecules, gene therapy, stem cell transplantation, tissue engineering, and reprogramming of cell and tissue types (amply quoted in [36, 46, 47]). These last approaches, that is, *cellular and tissue reprogramming*, are emerging as new and promising interventions [48]. They are based on de novo generation of cells, which may be obtained by converting adult somatic cells to pluripotent state termed *induced pluripotent stem* (iPS) cells (abovementioned) in vitro [48]. This cellular technology has been precisely defined *cellular reprogramming*, which has created new opportunities in understanding human disease, drug discovery, and regenerative medicine [49]. Today, the researchers are proposing a new generation or better a “second generation” of the cellular reprogramming [49]. It involves lineage-restricted transcription factors (as identified by Takahashi and Yamanaka in 2006 and defined Yamanaka factors [50]), or microRNAs able directly to reprogram one somatic cell to another (amply quoted in [51]). This “new version” of cellular reprogramming is based on the gene networks, which have been evidenced to be active during the development, and able to induce global shifts in the epigenetic landscape driving the cell fate decisions [49, 51]. The major advantage in the use of the direct reprogramming stays in controlling the resident support cells of adult stem cell niches inside eventual damaged organs or old tissues. This can facilitate the regeneration or repair of the lost or old tissue, by converting them into the desired cell type in situ [49, 51].

Interesting results on the use *in vivo* of reprogramming by forced expression of Yamanaka factors have been recently demonstrated in mice. Precisely, it has been observed that the induction of Oct4, Sox2, Klf4, and c-Myc transcription factors determined the detection of Nanog-positive cells (i.e., pluripotent cells) in diverse tissues, including the stomach, intestine, pancreas, and kidney [52]. Thus, reprogramming can be obtained *in vivo*. Furthermore, ulterior support derives from mouse bone marrow transplantation experiments and the detection of circulating iPSCs, suggesting that hematopoietic system may be reprogramed *in vivo* [52]. However, Abad and colleagues also demonstrated that Yamanaka factors induce a high grade of morality in mice [53]. The reasons prevalently are associated with the development of teratoma in multiple tissues, but they also hypothesize that the loss of cellular identified upon differentiation could impair organ or tissue functionality, resulting in organism death. Certainly, this may constitute a significant obstacle for applying it as regenerative or antiageing strategy. Alternatively, the partial reprogramming or the reprogramming of specific organ or tissue might be used. In these cases, the duration of the partial reprogramming must be regulated and potentially adapted to a specific cell, tissue, or organ. Consequently, it is possible to affirm that de novo generation of human cells remains elusive. Thus, many questions remain to be resolved. However, these approaches may one day help to ameliorate ageing symptoms, reduce the associated diseases, and ultimately improve human health and lifespan (amply quoted in [46, 52]).

As an alternative approach, *the use of young blood* appears surprising. It is based on research into the effects of *parabiosis*, tested in experimental mouse studies. It consists in connecting the circulatory systems of two organisms of different ages. These *heterochronic parabiosis's experiments* have elegantly demonstrated that exposing old tissues and organs to a young circulatory environment can rejuvenate tissue-specific stem cells, leading to a youthful state, which is characterized by functional and regenerative improvements [52, 54]. These encouraging data have led researchers from Stanford University to develop and perform a spinout, *the Alkahest*, as treatment for Alzheimer's disease, where the Grifols company, the largest plasma manufacturer worldwide, has invested for developing *Alkahest*'s plasma-based products. In addition, *heterochronic parabiosis experiments* have recently demonstrated the revitalization of brain function in old mice [55].

## 6. Nutritional Recommendations as Another Innovative Antiageing Approach: The Opening to Precision Nutrition Science

The diet is emerging as another modifiable risk factor for ageing and ARDs. Precisely, a diet low in vegetables, seafood, grains, nuts/seeds, and milk and high in red meat represents a strong risk factor [56]. Its weight is confirmed by studies, where nutritional practises have shown to decrease the risk for CVDs by 60% [57] and to prevent cancer for 27–39% by improving diet, physical activity, and body composition [58]. Ulterior support may derive from population-based approaches that can favourably shift disease risk factors in the entire population (amply quoted in [59]). However, these approaches may reveal vain because of several determinant factors: (1) the population's knowledge about the risk is generally insufficient to change behaviour, even if the vast majority of people know what they should and should not eat; (2) in addition, people have a limited amount of mental energy required to resist temptation; (3) it is now becoming clear the concept on existence of an interindividual variability in the effects to diet interventions (amply quoted in [59]). Thus, some dietary interventions may ameliorate the health of some individuals or not of others, being dependent on their genotypes, phenotypes, general clinical conditions, and environment (amply quoted in [59]). Accordingly, the major number of large, randomized controlled trials have demonstrated a successful response to diet interventions only in 40% of individuals [60]. This has suggested that the biological effects of dietary interventions differ between individuals (amply quoted in [59]). The determinant factors able to influence them are as follows: sex, age, habitual dietary habits, genetics, epigenetic, and gut microbiota [61]. This last modulates the absorption, distribution, metabolism, and excretion of compounds and metabolites [62, 63]. Thus, gut microbiota may affect bioavailability and biological responsiveness, as demonstrated in some studies [62, 63].

These observations and relative considerations have pioneered the introduction of a new promising medicine branch, the *Precision Medicine*, in order to overcome the interindividual variability in response to therapies [64]. The opening to this new medicine's vision has led to plan some trials with the focus on individual response to therapies, such as dietary interventions, which are now recognized by regulatory agencies, such as the US Food and Drug Administration. Thus, the precision nutrition approaches may affect the way to perform dietary interventions in the future [59]. Other factors that might affect the success of the implementation of precision nutrition approaches and would be consequently determined and included in the future studies are the metabolomic factors and the role of nutrigenetics in the interindividual responses to food components and products [59]. In addition, the key role of gut microbiota in the interindividual responses is becoming to include the gut microbiota analysis of each individual as a method for personalized dietary recommendation [65].

In complex, these observations point out the necessity to apply precision nutrition approaches for personalizing ageing and disease risk and, in parallel, future dietary recommendations. Further developments in this area will depend on considering big data analysis and health informatics that can capture molecular and medical data, as suggested above. In addition, the triumph of precision nutrition approaches will depend on the robust application of appropriate study designs as the predictive role of clinical individual biomarkers cannot be definitively ascertained without randomly assigning subjects to some form of control treatment [59–61].

### 6.1. Microbiota and Microbiome: Friends and Enemies in Different Periods of Life, Likely with Antagonistic Pleiotropic Effects?

As largely abovementioned, *microbiota and microbiome* are object of large interest in both ageing and ARD investigations, because of their crucial role in modulating diverse mechanisms and processes linked to both human health and diseases. For facilitating their understanding in the contest of this dissertation, it is well to define *microbiota and microbiome*. *Microbiota* represents an essential component of our body, constituted by a large panel of microorganisms (i.e., bacteria, virus, and mycetes) and in a number of about 3.8 × 10^13^ (amply quoted in [66]). It resides in different anatomical structures of the human body, organized in niches. However, it principally stays in the gut and precisely in the colon and consequently referred as *gut microbiota* (GM) [67, 68].

The term *microbiome* indicates the genome of microbiota, composed by a gene pool of about 150 times larger than that of the host (amply quoted in [66]).

GM creates a symbiotic relationship with the host (amply quoted in [66]). However, individual environmental and genetic factors can induce changes in its composition. In the same time, the host physiology is influenced by GM, by adapting to its presence and status. For example, it is well documented its influence on the immune gut component, as well as on immune/inflammatory pathways expressed in all tissue and organs, such as Toll-like receptor pathways (amply quoted in [66]).

The GM composition is usually created in early childhood and is modulated by several factors, including geographical factors, type of delivery, breastfeeding, age of weaning, antibiotic exposure, and dietary regimens [67, 68]. Among these, the diet and geographical location have, however, a key role in modulating GM composition. In fact, they are responsible of the interindividual GM heterogeneity, as demonstrated by several studies [67, 68]. GM mature composition is precisely achieved at the age of three years. It is relatively stable during the lifespan of an individual. However, adverse events, such as moderate lifestyle changes, acute diseases, and antibiotic treatments, can induce several changes [67, 68].

As mentioned, the diet and geographical location are the principal factors, influencing the interindividual heterogeneity observed in GM composition [66–68]. For example, high-fat diets have been associated with adverse effects on GM. Precisely, they generally induce a reduction in the representation of *Bacteroidete*s and an overgrowth of *Firmicutes*, including a wide range of opportunistic pathogens. The modifications at the level of order/phylum are referred as *dysbiosis* [66–68].These changes improve gut mucosa permeability and support systemic inflammation, subclinical immune activation, and metabolic alterations towards insulin resistance [66–68]. In this context, the relative ratio between *Bacteroides* and *Prevotella* has been suggested as a biomarker of healthy and active ageing, diet, and lifestyle [51]. Differently, several studies have also recently demonstrated that *Mediterranean diet* is associated with beneficial effects on GM, including higher biodiversity, overrepresentation of *Prevotella*, and underrepresentation of opportunistic pathogens [66–68].

Dysbiosis is remarkable in old age, characterized by consequent and negative modifications in GM functionality and relative stability [66–68]. As mentioned, it is characterized by a lower *Firmicutes*/*Bacteroidetes* ratio in the elderly (aged 70–90 years) and age-related modifications in subdominant microbiota, particularly represented by an increase in facultative anaerobes, including *Streptococci*, *Staphylococci*, *Enterococci*, and *Enterobacteria* [66–68]. Antibiotic treatment, hospitalization, and *Clostridium* difficile related to diarrhea (CDAD) associated with old age also contribute to the increase of *Enterobacteria*. Furthermore, a commonly accepted ageing effect magnified by antibiotic treatment, hospitalization, and CDAD is also the decrease in *Bifidobacteria* in terms of both abundance and species diversity [66–68]. Remarkable discrepancies in the behavior of *Bifidobacteria* have, however, been evidenced with respect to ageing, recently explained by country-related differences as well as the remarkable temporal instability of the *Actinobacteria* (the phylum that includes the Bifidobacterium genus) population in the fecal microbiota of the elderly [66–68]. Age-related GM modifications seem to start after a subject-specific “threshold age” influenced, indeed, by individual characteristics, such as diet and geographical location. Age-related dysbiosis can determine not only several inflammatory gastrointestinal diseases (i.e., gastrointestinal bleeding, infections, gastric and colon cancers, and constipation) in elderly, but also it can contribute to the onset of several ARDs, such as obesity, metabolic syndrome, diabetes, CVD, Alzheimer's disease, infection, and cancer [66–68]. One of the major causes at the base of the onset of these ailments (all with a chronic inflammatory pathophysiology) is probably the lipopolysaccharide (LPS) that triggers the secretion of proinflammatory cytokines at both systemic and gut tract levels [66–68]. LPS is continuously produced in the gut through lysis of Gram-negative bacteria and absorbed into intestinal capillaries to be transported by lipoproteins. Changes in GM could, therefore, be responsible for increased endotoxemia, which in turn would trigger the development of several age-related inflammatory pathologies. Thus, dysbiosis itself represents a possible ARD cause, because it is a continuous source of antigenic stimulation (i.e., LPS), able to deregulate immunity, and contributes to immunosenescence, and consequently, it is responsible for frailty. At the same time, recent data underline that ageing is the principal cause of dysbiosis. Ageing seems relatively to have little effect on the overall gastrointestinal function but, due to the decreased adaptive capacity of gastrointestinal tract, elderly people may not recover from illnesses or injuries as quickly as young adults. Decreased adaptive capacity of gastrointestinal tract may also reduce tolerability of medications for elderly. Other age-related factors, such as changes in diet, lifestyle, and GALT immunosenescence, dramatically influence the human gut ecosystem [66–68].

The administration of probiotics and/or prebiotics to elderly is, hence, reported to induce changes in several immune and inflammatory parameters, demonstrating that manipulation of GM may result in modification of functionality of an aged immune system (amply quoted in [66]). Even though the possibility of keeping immunosenescence and inflamm-ageing under control by a simple supplementation and/or functional food is interesting, the concept of “*immuno-nutrition*” is still immature and needs to be better related to the health and immunological and nutritional status of the elderly, as well as their nationality and actual age (amply quoted in [66]). Furthermore, the eventual “improvement” in immune and inflammatory status of elderly involved in feeding trials needs to be better defined, in terms of a true health advantage. Indeed, actually only a shorter duration of common infectious diseases has been reported as a positive effect of a probiotic supplementation, but not a decrease in infection incidence. Furthermore, our considerations regarding the application of pro-/prebiotics in distinctive intestinal conditions of the elderly (i.e., constipation and CDAD) underscore the necessity to enhance the very limited clinical evidence confirming its efficacy in prevention and especially treatment of these pathologies (amply quoted in [66]).

## 7. Physical Activity as Another Beneficial Antiageing/Anti-Inflammation Intervention

It is well documented in literature that habitual physical activity has several advantageous effects on human health, while its reduction has demonstrated to be one of the strong and independent factors associated with morbidity and mortality [69]. Among the beneficial effects, it is well recognized that exercise can positively modulate not only the life expectancy but also it can delay, retard, or prevent several ARDs, improving health-span for consenting likely to achieve a prolonged existence in health [70, 71]. In particular, physical activity has been demonstrated to reverse or attenuate the progression of tissue and organ ageing (i.e., the brain and cardiovascular system), since it induces positive vascular, structural, and neuromolecular changes. These last delay or retard insulin resistance, inflammation, and oxidative stress, all age-related conditions associated with vascular ageing and remodeling, CVDs, cognitive decline, and brain-related diseases [72, 73].

The beneficial or dangerous effect reciprocally mediated by active or reduced physical activity is the result of epigenetic factors [74], which modulate expression of genes codifying several pathways. For example, physical activity provokes insulin sensitivity and glucose disposal modulating the expression of pathways associated with inflammation and oxidative stress, which are high-risk factors for ageing and ARDs [75, 76]. In old individuals, independently by gender, the physical exercise reduces systemic inflammation, by decreasing the serum levels of typical inflammatory biomarkers, such as C-reactive protein, IL-6, and TNF-*α* (amply quoted in [77]). Accordingly, it has been demonstrated that in peripheral blood mononuclear cells from aged individuals, the exercise training reduces protein expression of TLR-2 and TLR-4 [78]. Similarly, physical exercise also seems to improve the circulating levels of endothelial progenitor cells (EPCs), cells related to both endothelial turnover, cardiovascular repair and regeneration, and adult angiogenesis [18]. Thus, physical exercise is associated with a good health of endothelium, an essential component of stroma of all tissues and organs [18]. Its dysfunction has been detected to contribute to the onset of the major number of ARDs, CVDs, and neurodegenerative diseases included, as amply described in our recent studies [13, 18].

Other accumulating data suggest that exercise is beneficial for human health in all periods of life only if it is of moderate intensity. In this case, it has health-promoting effects that are systemic and complex, undoubtedly involving regulation of redox homeostasis, inflammation, and related signaling, as described above [79]. In general, physical exercise causes an elevated generation of reactive oxygen species (ROS). ROS are important modulators of muscle contraction, antioxidant protection, and repair of damage from oxidation, all of which at moderate levels generate physiological responses. Several factors involved in mitochondrial biogenesis are modulated by exercise-associated redox changes. Certain endogenous thiol antioxidants, such as glutathione and thioredoxin, are modulated with exercise-related high oxygen consumption and ROS generation and control cellular function through redox-sensitive signaling and protein interactions. ROS may also play a role in exercise-induced angiogenesis, by inducing EPC mobilization. Also, exercise-related ROS production may be related to DNA methylation and histone modification, thereby creating heritable conditions controlled by epigenetics [79].

Based on these observations, it is possible to affirm that the regular physical activity of moderate intensity can be recommended as a beneficial antiageing/anti-inflammatory intervention. However, its effect appears to contribute to the maintenance of physiological conditions if synergically applied with nutritional interventions or other changes of lifestyle.

## 8. Considerations and Suggestions on the Treatments Described

**Figure 3:**
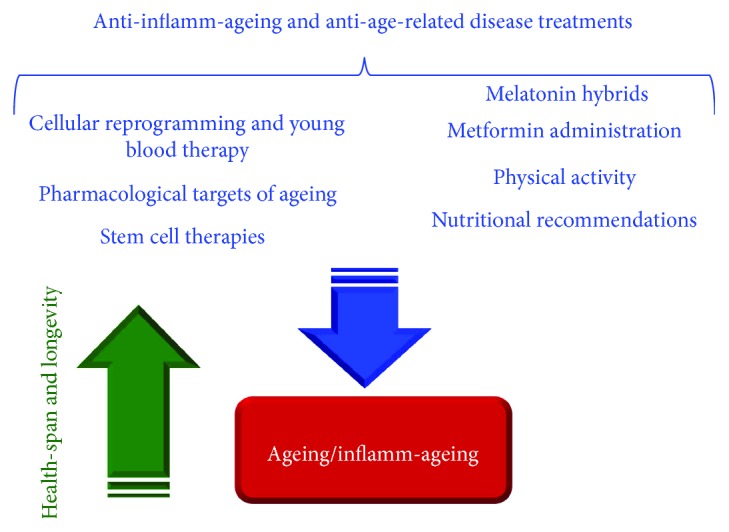
Anti-inflamm-ageing and anti-age-related disease treatments: described and discussed. They seem to have beneficial effects regarding the ageing/inflamm-ageing processes and reciprocally improving health-span and longevity.

Current evidence sustains a large grade of plasticity in cellular and organism ageing, and it is becoming to consider ageing as a set of pathologies. Accordingly, the manipulation of specific signaling pathways (e.g., insulin/IGF-1, mTOR, AMPK, and sirtuins) and particularly of immune inflammatory pathways, such as TLR-4, has demonstrated/is detecting the possibility to modulate both the ageing process and the onset/progression of ARDs in complex organisms (see Figures 1 and 2) [13–19]. In addition, heterochronic parabiosis experiments have elegantly demonstrated that exposing old tissues and organs to a young circulatory environment can rejuvenate tissue-specific stem cells, leading to a youthful state, which is characterized by functional and regenerative improvements [52, 54]. These encouraging data in animal models have given an optimistic hope to people in definitively achieving the *long life elixir*, considered a dream and a vanity's sin for a long time. Cotemporally, they are leading several ageing research groups to assure to obtain the same results in humans, developing several antiageing approaches, ranging from pharmacological targeting of ageing and basic biological assays to big data analysis, computational evaluations, the recent use of young blood, stem cells, cellular, genetic, and epigenetic reprogramming, or other techniques of regenerative medicine (see Figure 3) [1–8]. But, as evidenced in this brief overview on the emerging antiageing treatments, they are for the major number of more anti-inflamm-ageing and anti-ARD interventions than pure antiageing treatments, in agreement to current concept of ageing as disease's syndrome and the suggested Gems's definition of antiageing treatment, as stressed above [9, 10].

In addition, another crucial consideration emerges by their dissertation and their experimentation more in animal models than in humans. On the other hand, the major number of genes, molecules, and pathways associated with ageing and longevity has been detected in animal models and precisely in simple animal organisms [2–8]. As a consequence, only a small number of these genes, proteins, and pathways exist in human as homologs, but these are very rare, and consequently, our understanding on these molecules remains largely unknown [2–8]. This leads to insignificant discoveries. For example, pathways associated with longevity in simple animal models, such an IGF-1 pathway, often reveal irrelevant in human [2–8]. Consequently, the major open question is how antiageing interventions can reveal effective in humans. In order to achieve this goal, a possibility can derive from the use of big animals in the ageing experiments, including dogs [80] and primates [81]. But they show other problems for which the solutions are much less obvious. Firstly, large animal models are very, and sometimes, prohibitively expensive. In addition, they need a very intensive working setting, which requires a large and multidisciplinary staff and extended time cares. As a consequence, simple animal organisms appear until now the best animal model in ageing study, because they are relatively small, easy to handle, and not expensive. In addition, they can be genetically manipulated to mimic human diseases, by generating “knockout” or “knock-in” models and increasing their longevity in a rapidly progressing field (amply quoted in [46]).

Furthermore, most ageing-related genes and pathways have not yet been pharmacologically targeted. Computational studies might be developed, and they might help to identify and rank new modulator candidates and assemble biological insights obtained, as reviewed in our recent reviews and reported above. Such approaches are fundamental, as well as the integration of different types of investigations, as reported above. This reflects the relevant concept that each individual is the result of the sophisticated interplay between environmental factors and its genome, trascriptome, proteome, metabolome, microbiome, epigenome, and exposome [13–19]. Thus, it is necessary to perform a more complex combination of investigations based on genetic, transcriptomic, proteomic, metabolomic, microbiomic, and epigenetic evaluations, for obtaining interesting data in the study of ageing and ARDs. In addition, computational investigations are also recommended, as reported above, as well as collecting environmental and biometric data, medical/scientific/health care records. Thus, the integration of all data, obtained in this large panel of investigations, and their elaboration might lead to the development of appropriate agonists, antagonists, inhibitors of specific signaling ageing, and disease pathways, which might be used in the near future as personalized treatments for ageing and ARDs, facilitating their management and outcome [13–19]. On the other hand, this new vision of individual, as summary of environmental factors and its genome, trascriptome, proteome, metabolome, microbiome, epigenome, and exposome, also reflects the failure of the major number of antiageing interventions performed [13–19]. In fact, an interindividual variability of response among individuals, or better, among organisms enrolled in these investigations has been discovered. This, among other factors, has led to both the introduction of new branch of medicine, *the precision medicine* in order to overcome the interindividual variability in the response to therapies, and the goal to develop *personalized (tailored) therapies* [59, 64].

Thus, the capacity of scientific community to promote effective antiageing interventions in humans, which after are evaluated in clinical trials, until now remains reduced. This evidences another limitation, the long time required for the clinical validation needed to convert or translate the interventions proposed and analyzed in effective therapies and to obtain the regulatory approval. In fact, clinical trials are long and expensive, and the success of clinical trials performed until now remains very low. In line with these observations, interesting and promising might be the development of clinical trials having the goal to evaluate the effects of metformin in people without diabetes and at different ages or the consumption of foods rich in melatonin or melatonin hybrids, as well as to investigate the use of young blood therapies, senescent cell ablation, moderate physical activity, precision nutrition interventions, and nutraceuticals associated with the analysis of the composition and status of GM in everyone.

## 9. Concluding Remarks and Future Perspectives

Multidisciplinary approaches, as well as their integration and elaboration of big data, are fundamental in the vision to develop effective antiageing therapies and recommendations. In addition, the antiageing therapies developed and proposed until now by several biotechnology companies that appear very expensive *much lead us a large reflection*, as well as the *dishonourable tacit complicity* of doctors, laboratories, and companies in proposing palliative cares and not the desired long life elixir. The solution might be the goal of ageing and ARD research in order to make new discoveries, although, the ways to execute still are long and difficult. On the other hand, Marcel Proust affirmed “*The real voyage of discovery consists not in seeking new landscapes, but in having new eyes.*”
